# The utility of a composite endpoint for tracking disease progression in Lewy body dementia

**DOI:** 10.1002/trc2.70260

**Published:** 2026-05-20

**Authors:** Elie Matar, Simon R. White, John‐Paul Taylor, Alan Thomas, Ian McKeith, Joseph Kane, Simon Lewis, Ajenthan Surendranathan, John O'Brien

**Affiliations:** ^1^ Sydney Medical School Faculty of Medicine and Health University of Sydney Sydney New South Wales Australia; ^2^ Brain and Mind Centre Faculty of Medicine and Health University of Sydney Sydney New South Wales Australia; ^3^ Department of Neurology Royal Prince Alfred Hospital Camperdown New South Wales Australia; ^4^ Department of Psychiatry University of Cambridge School of Clinical Medicine Cambridge UK; ^5^ MRC Biostatistics Unit University of Cambridge Cambridge UK; ^6^ Newcastle Translational and Clinical Research Institute Campus for Ageing and Vitality Newcastle University Newcastle upon Tyne UK; ^7^ Centre for Public Health Queen's University Belfast Belfast UK; ^8^ Parkinson's Disease Research Clinic Macquarie University Sydney New South Wales Australia; ^9^ Department of Neurology University College London Hospital London UK

**Keywords:** caregiver burden, clinical trials, composite endpoints, disease progression, Lewy body dementia, multidomain assessment, outcome measures, Parkinson's disease dementia

## Abstract

**INTRODUCTION:**

Dementia with Lewy bodies (DLB) or Parkinson's disease dementia (PDD), collectively termed Lewy body dementia (LBD), show heterogenous progression across cognitive, motor, and neuropsychiatric symptom domains, yet disease‐specific endpoints are lacking. We evaluated whether a composite clinical endpoint using validated scales across different symptom domains could sensitively track disease progression and align with functional and caregiver outcomes.

**METHODS:**

One hundred sixteen participants (DLB = 72; PDD = 44) were assessed at baseline, 3, and 6 months in a cluster‐randomized trial comparing usual care versus management informed by an evidence‐based toolkit. The Lewy Body Symptom Severity (LBSS) index was constructed by summing rescaled Mini‐Mental State Examination, Movement Disorder Society Unified Parkinson's Disease Rating Scale (Part III), Dementia Cognitive Fluctuations Scale, and Neuropsychiatric Inventory 4‐item subscore (including hallucinations). Linear mixed‐effects models tested change over time. Validity was examined against caregiver Clinical Rating of Change (CRC), Bristol Activities of Daily Living (ADL) Scale, and caregiver Zarit Burden Interview.

**RESULTS:**

Over 6 months, LBSS increased significantly (*β* = 0.0307; *P* = 0.0006). Simulation‐based power analyses indicated greater statistical efficiency for LBSS than for any individual component. LBSS also detected a significant intervention effect (*P* = 0.0365) not observed with single‐domain measures. LBSS correlated with caregiver burden (Zarit; *ρ* = 0.53, *P* < 0.001), functional dependence (Bristol ADL; *ρ* = 0.57, *P* < 0.001), and CRC (*ρ* = −0.33, *P* = 0.002), permitting derivation of a minimal clinically important difference.

**DISCUSSION:**

A simple composite spanning cognition, parkinsonism, cognitive fluctuations, and neuropsychiatric symptoms sensitively detected short‐interval progression, with improved statistical efficiency over single‐domain measures, and was aligned with functional/caregiver outcomes. These findings support composite endpoints for LBD trials and can inform the design of disease‐specific scales.

## Highlights

- A multidomain composite index detects 6‐month progression in Lewy body dementia.
- The composite shows greater statistical efficiency than individual domain measures.
- Composite change correlates with functional dependence and caregiver burden.
- Simulation‐based analyses estimate reduced sample sizes for longitudinal trials.
- A pragmatic framework is presented for multidomain endpoints in heterogeneous Lewy body dementia.

## 1 INTRODUCTION

Lewy body dementia (LBD), which includes dementia with Lewy bodies (DLB) and Parkinson's disease (PD) dementia (PDD), represents the second most common form of neurodegenerative dementia after Alzheimer's disease (AD). LBD has long been recognized as a heterogenous syndrome characterized by a distinct constellation of cognitive, motor, and neuropsychiatric symptoms.^1^, ^2^ Alongside cognitive impairment, current diagnostic frameworks incorporate a combination of core clinical features including fluctuating cognition, recurrent visual hallucinations, parkinsonism, and rapid eye movement (REM) sleep behavior disorder^2^ Recent work has highlighted the heterogeneity of symptom manifestation and progression in LBD, which may reflect distinct clinical and biological subtypes.^3^, ^4^, ^5^ Previous work has shown that presenting and dominant features may differ and indeed cluster across patient cohorts with suggestions of motor‐, cognitive‐, or even neuropsychiatric‐dominant presentations, which may progress differently throughout the disease course.^4^, ^6^ Recent natural history studies have also confirmed significant differences in the timing and onset of core features of DLB between patients, with differences also occurring between sexes.^7^

This multidomain heterogeneity poses a particular challenge both for assessing disease progression and for outcome measurement in clinical trials for LBD. Most interventional studies for LBD have adopted single‐domain endpoints (e.g., cognitive or motor scales) or global impression ratings developed for other dementias.^8^ Meanwhile, multidomain instruments developed for PD, such as the Movement Disorder Society Unified Parkinson's Disease Rating Scale (MDS‐UPDRS),^9^ have been applied in LBD but remain weighted toward motor‐related disability, with key features of LBD, such as cognitive fluctuations, not explicitly represented. These measures may therefore fail to detect meaningful change when deterioration is confined to non‐targeted domains or when improvements in one area offset declines in another. As a result, trials risk underestimating treatment effects or progression rates, particularly over the short intervals that are common in phase 2 and 3 designs. With the emergence of disease‐modifying therapies (DMTs), there is a pressing need for global outcome constructs that are sensitive to changes across differing axes of progression. A systematic review of LBD trials (up to 2022) showed that more than half were testing DMTs.^10^ The urgency is heightened by recent successes with amyloid‐targeting therapies in AD^11^ and synuclein‐targeting therapies in PD,^12^ which are likely to have translational applicability to LBD.

**RESEARCH IN CONTEXT**

- **Systematic review**: Clinical trials in Lewy body dementia (LBD) have predominantly relied on single‐domain outcomes or global impression scales, despite marked heterogeneity across cognitive, motor, neuropsychiatric, and fluctuation symptoms, highlighting a need for outcome measures that better capture multidomain disease progression. While composite endpoints are increasingly used in Alzheimer's disease and other neurodegenerative conditions to improve sensitivity and efficiency, their application in LBD has not been systematically evaluated.
- **Interpretation**: Using prospectively acquired data from a multicenter randomized trial, this study demonstrates that a simple multidomain composite index can sensitively detect short‐interval progression in LBD, correlate with functional dependence and caregiver burden, and offer greater statistical efficiency than individual domain measures. These findings suggest that composite endpoints may better capture overall disease burden in heterogeneous LBD populations.
- **Future directions**: These findings support further validation and refinement of composite endpoints for LBD, including evaluation across independent cohorts and in interventional trials, to complement emerging disease‐specific outcome measures.

Composite clinical endpoints, which integrate validated measures from multiple domains, offer a potential solution for clinical trials.^13^ Such approaches have shown particular relevance in AD, in which composite scores have proven effective at detecting rates of change and are now widely used.^14^ Similar approaches have been adopted in other neurodegenerative conditions including Huntington's disease^15^, progressive supranuclear palsy,^16^ and frontotemporal dementia.^17^, ^18^ Whether similar tools would be useful in DLB has not been systematically evaluated. An optimally designed composite should demonstrate sensitivity to progression, correlate with functional outcomes and caregiver burden, and retain feasibility for use across trial sites.

We have previously shown that validated scales for different domains of Lewy body disease can adequately measure progression of LBD over a time period compatible with clinical trials^19^. In this study, we aimed to assess the utility of a multidomain composite in tracking short‐interval disease progression in LBD. Using prospectively acquired data from a multicenter cohort, we evaluated a composite incorporating cognition, motor function, cognitive fluctuations, and neuropsychiatric symptoms. In this cohort, the intervention arm consisted of administering a management toolkit, which ideally should comprise expected standards of practice, thus being feasible as a naturalistic study in which the progression of clinical features can be observed and modeled. Our objectives were to (1) determine whether the composite detects progression over 6 months, (2) examine its validity against functional dependence and caregiver burden, and (3) compare its statistical power to individual component measures. Our hypothesis was that a composite can capture overall disease burden, reflect the lived experience of patients and caregivers, and reduce sample sizes required to detect change.

## 2 METHODS

### 2.1 Clinical cohort

Data from 127 subjects with LBD (77 DLB, 50 PDD) were analyzed from a cluster randomized controlled trial—Diagnosis And Management Of Neurodegenerative Dementia (DIAMOND‐Lewy Study; ISRCTN11083027)—conducted between 2016 and 2017 in 23 memory or movement disorder services across eight National Health Service (NHS) jurisdictions in the UK.^20^ All patients underwent clinical assessment and were diagnosed as having probable DLB or PDD according to consensus criteria in use at the time.^1^, ^21^ Half of the services were randomized to receive a management toolkit summarizing current evidence‐based guidelines for symptomatic treatment of LBD (freely available from: https://research.ncl.ac.uk/diamondlewy/managementtoolkit/), while the other half continued with standard care (control arm). From these services, 131 subjects were recruited for assessment at baseline, 3 months, and 6 months. Of the 131 recruited, 127 participants underwent baseline assessments. For inclusion in the present analysis, data for at least two timepoints were required to contribute to group‐level progression models. Participants lost to follow‐up after baseline (*n* = 11) were excluded, yielding 116 participants (72 DLB, 44 PDD) for the final analysis. These comprised 107 with data at all three timepoints (66 DLB, 41 PDD), 7 with baseline and 3‐month data, and 2 with baseline and 6‐month data. Ethical approval for this study was obtained from the West Midlands–Coventry and Warwickshire Research Ethics Committee (reference 16/WM/0025).

### 2.2 Consent statement

Written informed consent was obtained from all participants with capacity and from all caregivers for their own participation. For participants lacking capacity, assent was obtained from caregivers in accordance with ethical approval.

### 2.3 Clinical assessments

Assessments at baseline, 3 months, and 6 months were conducted by the same trained research team members within each region, with assessors blinded to the service allocation. Cognitive function was measured using the Mini‐Mental State Examination (MMSE; range 0–30, higher scores indicating better cognition). The MMSE was selected as the cognitive component as prior analyses of this cohort demonstrated greater sensitivity to short‐interval change compared to the Montreal Cognitive Assessment.^19^ Cognitive fluctuations were assessed with the Dementia Cognitive Fluctuation Scale (DCFS; range 6–30, higher scores indicating greater fluctuation severity).^22^ Neuropsychiatric symptoms were measured using the Neuropsychiatric Inventory (NPI; range 0–144, higher scores indicating greater symptom severity), which rates 12 behavioral domains from a structured interview with the caregiver^23^. From the NPI, the NPI‐4 (sum of hallucinations, delusions, depression, and apathy) was derived. The NPI‐4 has previously been shown to be sensitive in LBD and has been used as a primary endpoint in prior randomized trials.^24^ Motor parkinsonism was evaluated using the (MDS‐UPDRS III; range 0–132, higher scores indicating more severe motor impairment).^9^, ^25^ Measures of functional dependence (Bristol Activities of Daily Living Scale,^25^ higher scores indicating greater dependence) and caregiver burden (Zarit Burden Interview^26^, higher scores indicating greater caregiver burden) were also collected. Caregiver‐reported global Clinical Rating of Change (CRC) was assessed at 3 and 6 months and prespecified as the anchor measure for assessing clinical meaningfulness and criterion validity of the composite, given its ability to reflect change in everyday cognitive, behavioral, and functional status across domains.

Medication class and dosage were recorded at each visit. Over the course of the trial, three patients (2.6%; all in the control arm) had changes in the type or dose of antipsychotic medication, 20 patients (17.2%; 8 in the control arm and 12 in the intervention arm) had changes in dopaminergic medication, and 21 patients (18.1%; 13 in the control arm and 8 in the intervention arm) had changes in acetylcholinesterase inhibitor therapy.

### 2.4 Composite endpoint construction

The Lewy Body Symptom Severity Index (LBSS) was developed to capture the multidomain symptom burden characteristic of LBD and to align with established clinimetric principles. Conceptually, the LBSS can be understood in two complementary ways. From a reflective model perspective, disease progression in LBD can be considered one or more latent traits representing underlying neurodegenerative processes, which manifest as correlated changes in cognition, cognitive fluctuations, parkinsonism, and neuropsychiatric symptoms. From a formative model perspective, these domains also act as constitutive components that together define the construct of LBD symptom severity. The latter was the primary aim of the construct composition. Each domain was measured using a validated clinical scale: MMSE for cognitive impairment, DCFS for cognitive fluctuations, MDS‐UPDRS III for parkinsonism, and NPI‐4 for neuropsychiatric symptoms rescaled to range from 0 to 1 and summed to form the index:

$$\begin{array}{ccc} \mathrm{LBSS} & = & \left(\frac{30-\mathrm{MMSE}}{30}\right)+\left(\frac{\mathrm{UPDRS}-\mathrm{III}}{132}\right)\\ & & +\;\left(\frac{\mathrm{DCFS}-6}{30}\right)+\left(\frac{\mathrm{NPI}-4}{48}\right) \end{array}$$

To ensure commensurate directionality of components, MMSE was reversed so that higher values on each term reflected greater impairment; higher LBSS values therefore indicate greater overall symptom severity. Given the recognized heterogeneity of LBD and the absence of empirical evidence favoring one symptom domain, equal weights were applied across the four components to provide a simple, transparent, and clinically agnostic index. Equal weighting was specified a priori to avoid data‐driven optimization within a single cohort and to preserve interpretability across studies. This parsimonious specification minimizes assumptions and overfitting (see Discussion and Materials S1 in supporting information for alternative weighting strategies).

For sensitivity analyses, we also created a standardized version of the index in which each component was converted to a *z* score based on the cohort baseline measures (Materials S1). For clarity, in the main text we only present results of the simpler sum of rescaled scores above. All component measures were collected as part of routine clinical and research assessments at the time of data acquisition. The composite score is an analytic index derived from these measures rather than a proprietary instrument.

### 2.5 Statistical analysis

Analyses were conducted using Python (version 3.11) and the statsmodels package (version 0.14) for the primary modeling, and MATLAB (R2019b) for simulation‐based power estimation. Change in the LBSS over time was examined using linear mixed‐effects models with random intercepts for participants to account for repeated measures. Analyses were conducted using a complete‐case approach, with no imputation performed. Fixed covariates in all models included age, diagnosis (DLB or PDD), antipsychotic use, cholinergic medication use, and levodopa equivalent dose. Time was coded as months since baseline (0, 3, and 6). Identical models were fitted for MMSE, MDS‐UPDRS III, DCFS, and NPI‐4 to permit direct comparison to the index. The potential effect of the intervention was explored by including a group × time interaction term in the LBSS model.

Criterion validity was evaluated using Spearman correlations between LBSS and measures of functional dependence (Bristol Activities of Daily Living Scale) and caregiver burden (Zarit Burden Interview) across all timepoints. For power and sample size estimation, fixed‐effect coefficients and variance components from the pre‐specified linear mixed‐effects models (with random intercepts for participants) were used to generate simulated datasets in MATLAB across the three repeated measurement occasions (0, 3, and 6 months). For each sample size, 1000 replicate datasets were generated and analyzed. Power was defined as the proportion of simulations in which the fixed effect of time reached significance at *α* = 0.05, and the minimum sample size required for 80% power was estimated. All statistical tests were two tailed, with *P* < 0.05 considered statistically significant.

## 3 RESULTS

### 3.1 Baseline characteristics of the DIAMOND‐Lewy cohort

Baseline characteristics of the DIAMOND‐Lewy participants are demonstrated in Table 1. There were no differences in age or sex between diagnostic and intervention groups. Overall, participants with LBD predominantly fell within the mild‐to‐moderate dementia stage. There was no difference in proportion of patients in the intervention arm between diagnostic groups.^20^ The LBSS did not differ between diagnostic groups or intervention arms. As expected, the proportion of patients with parkinsonism and MDS‐UPDRS motor subsection scores (UPDRS III) were higher in PDD than DLB. There was no significant difference in the other constituent measures.

**TABLE 1:** Linear mixed‐effects analyses of longitudinal progression and intervention effects baseline demographic and clinical characteristics.

	DLB (*n*=72)	PDD (*n*=44)	LBD (*n*=116)
Age (years)	77.1 ± 7.1	79.2 ± 6.9	77.9 ± 7.2
Male, n (%)	55 (76.4)	36 (81.2)	91 (78.4)
Intervention group, n (%)	42 (58.3)	21 (47.7)	63 (54.3)
MMSE	21.7 ± 5.9	20.5 ± 6.6	21.5 ± 5.9
Parkinsonism, n (%)**	52 (72.2)	44 (100)	87 (75)
Visual hallucinations, n (%)	44 (61.1)	37 (84.1)	82 (70.7)
Fluctuations, n (%)**	45 (62.5)	15 (34.1)	60 (51.7)
pRBD	36 (50)	22 (50)	59 (50.8)
UPDRS‐III*	35.8 ± 16.5	48 ± 20.8	39.6 ± 18.5
DCFS total	13.1 ± 5.1	12.3 ± 4.1	12.7 ± 4.8
NPI‐4	10.7 ± 10.1	7.7 ± 6.4	9.5 ± 8.9
LBSS	0.92 ± 0.38	0.99 ± 0.42	0.94 ± 0.40
LED (mg)**	146 ± 209	484 ± 270	317 ± 295
ChI use, n (%)	55 (76.3)	29 (65.9)	84 (72.4)
Antipsychotic use, n (%)	11 (15.2)	2 (4.5)	13 (11.2)

Notes: Values displayed as mean ± standard deviation and percentage (number). Asterisks denote significant difference between DLB and PDD (two‐tailed independent‐samples *t* test).

Abbreviations: ChI, cholinesterase inhibitor; DCFS, Dementia Cognitive Fluctuation Scale; DLB, dementia with Lewy bodies; LBSS, Lewy Body Symptom Severity Index; LBD, Lewy body dementia; LED, levodopa equivalent dose; MMSE, Mini‐Mental State Examination Score; NPI, Neuropsychiatric Inventory; PDD, Parkinson's disease dementia; pRBD, probable rapid eye movement sleep behavior disorder; UPDRS‐III, Unified Parkinson's Disease Rating Scale (Section III).

*p < 0.01, **p < 0.001.

### 3.2 Composite score detects change over time

In the primary mixed‐effects model with random intercepts for participants and prespecified covariates (age, diagnosis, levodopa equivalent dose, antipsychotic use, cholinergic medication use), LBSS increased significantly over time (monthly change of *β* = 0.0307 [95% confidence interval (CI) 0.0133–0.0481], *p* = 0.0006), indicating progressive worsening of the combined sum of cognitive, motor, and neuropsychiatric symptoms across the cohort over the 6‐month follow‐up (see Table 2). Findings were directionally consistent in sensitivity analyses using alternative composite encodings comprising *z* scored constituent domain scores (Materials S1 and Table S1 in supporting information). Consistent with previous analyses^19^, analysis of individual domain trajectories with their respective covariates showed variable progression over time (Materials S2 in supporting information). Comparison of the composite LBSS against individual domains is presented below, finding superiority in relation to intervention effects and efficiency in detecting change over time.

**TABLE 2:** Mixed‐effects model estimates of longitudinal change and intervention effects for the LBSS and component measures.

Outcome	β	SE	*p* value	AIC	BIC
**Time**					
MMSE	−0.22	0.14	0.027	1179.7	1209.3
UPDRS	+0.82	0.32	0.010	2429.3	2462.7
DCFS	+0.29	0.10	0.003**	1135.9	1166.0
NPI‐4	+0.21	0.19	0.290	1364.9	1398.1
LBSS	+0.031	0.009	0.0006***	98.2	133.4
**Intervention x Time**					
MMSE	+0.09	0.19	0.626	1179.7	1209.3
UPDRS	−0.47	0.41	0.249	2429.3	2462.7
DCFS	−0.15	0.14	0.271	1135.9	1166.0
NPI‐4	−0.56	0.27	0.040*	1364.9	1398.1
LBSS	−0.025	0.012	0.036*	98.2	133.4

Notes: Linear mixed model estimates of longitudinal change (time) and intervention effects (intervention × time) for the LBSS and its components. β values represent fixed‐effect estimates of the slope (mean change per unit time) for each outcome, with associated SE and *p* values. “Time” reflects the average trajectory across all participants, while “intervention × time” represents the difference in slope between intervention and control groups. AIC and BIC are reported as measures of relative model fit (lower the better). Significance levels are indicated as *p < 0.05, **p < 0.01, ***p < 0.001.

Abbreviations: AIC, Akaike information criterion; BIC, Bayesian information criterion; DCFS, Dementia Cognitive Fluctuation Scale; LBSS, Lewy Body Symptom Severity Index; MMSE, Mini‐Mental State Examination Score; NPI, Neuropsychiatric Inventory; SE, standard error; UPDRS, Unified Parkinson's Disease Rating Scale.

### 3.3 Composite score detects effect of intervention

In the linear mixed models for LBSS, the interaction between intervention and time was statistically significant and negative (*β* = −0.0252 [95% CI: −0.0488 to −0.0016], *p* = 0.0365), indicating that participants in the intervention arm experienced a slower rate of symptom progression, as measured by the LBSS, than those receiving standard care. This effect was not detected for most individual domain measures, in which changes were directionally consistent but did not reach statistical significance (Table 2). These included a trend toward improvement in MMSE and reductions in DCFS and MDS‐UPDRS III scores, alongside a statistically significant interaction for NPI‐4.

### 3.4 Composite score is more efficient at detecting longitudinal change

**FIGURE 1:**
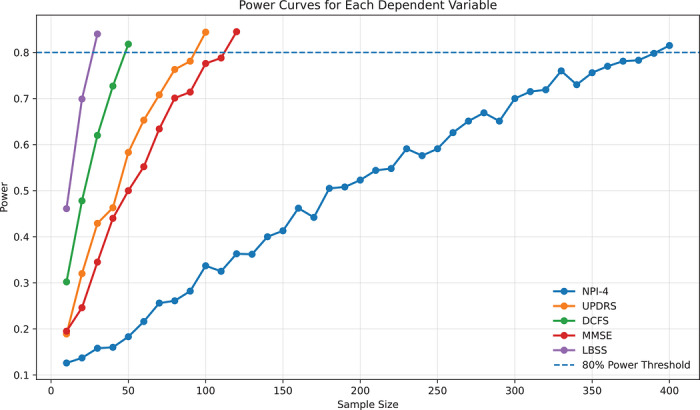
Monte Carlo simulation–based power analyses were performed to estimate the minimum sample size required to detect longitudinal change (fixed effect of time) in linear mixed‐effects models of candidate endpoints. Power curves are shown for NPI‐4, DCFS, LBSS, MMSE, and MDS‐UPDRS III (UPDRS) across increasing sample sizes. Each curve represents 1000 iterations at each sample size (increasing by a step size of 10 participants), with three repeated measures per subject (0, 3, and 6 months). Power increased near‐linearly before plateauing at larger cohorts. The dashed line indicates the 80% threshold for adequate statistical power. The composite LBSS achieved > 80% power at *n* = 30, while considerably larger cohorts were required for individual measures. DCFS, Dementia Cognitive Fluctuations Scale; LBSS, Lewy Body Symptom Severity Score; MMSE, Mini‐Mental State Examination; NPI‐4, Neuropsychiatric Inventory 4‐item subscore; MDS‐UPDRS III, Movement Disorder Society Unified Parkinson's Disease Rating Scale, Part III.

Monte Carlo simulation‐based power analyses were conducted to estimate the minimum sample size required to achieve 80% statistical power (*α* = 0.05) for detecting the fixed effect of time in linear mixed‐effects models for the composite and constituent scores. Across all outcome measures, the observed power increased in a near‐linear fashion (Figure 1). As an endpoint, the LBSS demonstrated greater statistical efficiency relative to its individual counterparts, achieving > 80% statistical power at a sample size of *n* = 30. In contrast, the individual components required considerably larger sample sizes to achieve equivalent (> 80%) power to detect a significant change over time: MMSE (*n* = 120), MDS‐UPDRS III (*n* = 100), DCFS (*n* = 50), and NPI‐4 (*n* = 400).

### 3.5 Convergent and criterion validity of the composite score

Across all available timepoints, LBSS showed strong positive correlations with caregiver burden as measured by the Zarit Burden Interview (*ρ* = 0.53, *p* < 0.001) and with functional dependence on the Bristol Activities of Daily Living Scale (*ρ* = 0.57, *p* < 0.001), demonstrating convergent validity against established measures of disease impact on patients and caregivers.

Criterion validity was examined using caregiver‐reported global CRC at 3 and 6 months. Change‐score analyses demonstrated that improvement in LBSS (decrease from baseline) aligned with better global ratings at both timepoints (3 months: *ρ* = −0.333, *p* = 0.002; *n* = 86; and 6 months: *ρ* = −0.234, *p* = 0.026; *N* = 91). Ordinal logistic models confirmed that change in LBSS (ΔLBSS) was associated with CRC category (per 1 standard deviation [SD] change: odds ratio [OR] = 2.36, 95% CI 1.49–3.73, *p* < 0.001 at 3 months; OR = 1.85 [95% CI 1.24–2.78], *p* = 0.003 at 6 months).

### 3.6 Minimal clinically important difference in LBSS

For illustrative purposes, an anchor‐based minimal clinically important difference for LBSS was estimated using the dichotomized caregiver‐reported global clinical impression of change scores at 3 and 6 months. When CRC was dichotomized (“improved” vs. “not”), mixed‐effects logistic models (random intercept by subject) indicated that each 1 SD decrease in LBSS increased the odds of being rated “improved” by ≈ 2‐fold (inverse ORs = 2.12 at 3 months and 2.14 at 6 months; original ORs per 1‐SD increase in LBSS = 0.471 [95% CI: 0.280–0.792], *p* = 0.0050, *n* = 95; and 0.466 [95% CI: 0.262–0.830], *p* = 0.010, *n* = 91).

Receiver operating characteristic analyses demonstrated fair discrimination of being rated “improved” by ΔLBSS (area under the curve [AUC] = 0.682 at 3 months; 0.663 at 6 months), with the Youden‐optimal thresholds of ΔLBSS = −0.13 (3 months; AUC 0.68) and −0.16 (6 months; AUC 0.66). We therefore propose a minimal clinically important difference in LBSS to be ≈ −0.13 (conservative −0.16 points), indicating that a decrease of at least this magnitude represents a clinically meaningful improvement in this cohort.

## 4 DISCUSSION

In this exploratory study using a large cohort of patients with LBD followed longitudinally in a randomized controlled trial setting, we evaluated the utility of a composite score derived from established clinical instruments. We were able to show that a simple construct (LBSS) comprising the linear sum of rescaled scores capturing key symptom domains of cognition, fluctuations, motor severity, and neuropsychiatric symptoms was able to detect clinically significant change over a 6‐month timeframe in a clinical trial. Importantly, the LBSS was more sensitive and statistically efficient than its constituent measures, achieving comparable power with markedly smaller sample sizes. The composite also covaried with indices of disease impact and caregiver‐reported global measures of change supporting its clinical validity. Notably, the LBSS detected an intervention effect that was not identified using single‐domain scales, suggesting that a unified multidomain measure may be more responsive than isolated symptom measures to treatments that modify overall disease burden.

The findings of this multicenter study give support to the concept that LBD severity at a group level may be better indexed by a multidomain measure capturing cognitive, motor, and neuropsychiatric domains. Historically, interventional trials in LBD have tended to emphasize a single domain for the primary efficacy endpoint such as cognitive outcomes (Alzheimer's Disease Assessment Scale Cognitive subscale,^27^ or MMSE,^28^, ^29^) neuropsychiatric symptoms (e.g., NPI, NPI‐4^30^, Scale for the Assessment of Positive Symptoms^31^, ^32^) or motor function (UPDRS‐III for parkinsonism^33^) often in conjunction with global impression scales as co‐primaries. Although broader clinical rating scales such as the MDS‐UPDRS capture several motor and non‐motor features, they are weighted toward motor‐related disability and were not constructed to provide a balanced composite index of overall disease burden in LBD. While these may be appropriate for symptomatic treatments targeting one feature, this approach may not be relevant to DMTs for which decline may occur variably across different but equally disabling symptom domains. This is appreciated across other multidomain conditions such as Huntington's disease, for which the composite Unified Huntington's Disease Rating Scale has shown advantages over motor or cognitive subscales alone^15^. While we await development of equivalent multidomain measures for LBD, composite indices have shown promise in other related disorders. In AD for example, the Integrated Alzheimer's Disease Rating Scale and the Alzheimer's Disease Composite Score were explicitly developed to improve sensitivity to progression in early disease and are now widely adopted in therapeutic development^34^.^14^, ^35^ Paralleling this work, the LBSS represents a first step toward creating a composite index tailored to LBD using validated scales.

In earlier analyses, we showed that the MMSE, MDS‐UPDRS III, and DCFS could detect significant change over 6 months.^19^ However, variance estimates for each individual measure were often of similar magnitude to the observed change, indicating marked inter‐individual variability and limiting sensitivity to detect clinically meaningful progression. Using the simplest combination of these features (the sum of scaled scores), the LBSS not only captured significant change over time, which supports it as a construct of disease progression, but did so with substantially greater statistical efficiency than single‐domain measures. Moreover, the average change in LBSS corresponded to an average increase of ≈ 0.18 points over 6 months, which was comparable to the estimated minimal clinically important difference (≈ 0.13–0.16) representing a modest but consistent multidomain worsening over a clinically relevant interval. Power analyses showed that the LBSS could detect statistically significant progression over time with fewer than half the participants required for the most efficient single‐domain measure. Such efficiency gains have substantial clinical implications in LBD, for which recruitment is challenging and trial attrition is high.

From a regulatory standpoint, a clinically meaningful endpoint in dementia is typically defined by evidence of an impact on daily functioning and caregiver burden.^36^, ^37^ In this regard, the LBSS correlated strongly with both functional dependence and caregiver burden, supporting its validity as an outcome measure. Accordingly, change in the LBSS reflects concordant change across multiple clinically relevant symptom domains rather than improvement in a single feature, aligning with how patients and caregivers experience overall disease burden. For point of illustration, we also used an anchor‐based analyses against global ratings of change to derive preliminary estimate of a minimal clinically important difference in the range of −0.13 to −0.16, providing an interpretable threshold. This threshold should be considered provisional and cohort specific, but in a trial context may be applied to define clinically meaningful change at the individual level or to support responder‐based analyses. These findings illustrate that a composite score can be both statistically responsive and clinically meaningful.

The DIAMOND‐Lewy trial, a feasibility study evaluating an evidence‐based LBD management toolkit in NHS services, originally found no significant effects of the intervention on most patient outcomes, though an impact was detected for reduced caregiver stress, reduced caregiver depressive symptoms, and a reduction in caregiver‐rated patient deterioration.^20^ However, in this secondary analysis the LBSS detected a significant attenuation of progression in the intervention arm, whereas individual domain measures showed heterogeneous responses. As this was not a prespecified analysis, the finding should be interpreted with caution. Nevertheless, the broader and more stable signal captured by the composite suggests that the LBSS may be more sensitive than individual measures in detecting treatment effects. This likely reflects both aggregation of change across multiple domains and improved signal‐to‐noise characteristics as reflected by reduced variability and greater statistical efficiency, providing evidence of incremental and construct validity. This supports the potential value of the LBSS as an outcome measure in future trials, particularly in heterogeneous cohorts in which benefits may emerge unevenly across domains.

Strengths of our study include the use of a large, multicenter cohort including both DLB and PDD participants recruited across routine NHS services; prospective longitudinal design with repeated assessments allowing reliable modeling of short‐term progression relevant to clinical trial timeframes; blinded assessments; and use of multiple outcomes beyond the composite allowing evaluation of content, construct and criterion validity, as well as derivation of a preliminary minimal clinically important difference. However, several limitations warrant consideration. The LBSS was constructed using established, validated clinical scales, with a priori selection of particular domains rather than data‐driven optimization. Furthermore, each component of the score was weighted equally. This pragmatic and transparent approach ensured clinical interpretability but may not fully capture differences in responsiveness or variance across domains. While appropriate for early‐phase validation, future work may incorporate empirically derived or stakeholder‐informed weighting to refine sensitivity (see Materials S1.5 for an illustrative example of data‐driven optimization). Because the composite combines rescaled scores across several domains, the clinical interpretability of absolute LBSS values at a single timepoint remains to be established, and the scale is currently best interpreted as a measure of longitudinal change. Additionally, while the LBSS demonstrated responsiveness over 6 months, its long‐term performance and cross‐cohort validity remain untested. Finally, as this analysis was conducted retrospectively within a clinical trial population with defined inclusion and exclusion criteria and predominantly included individuals with mild‐to‐moderate dementia, the findings may not fully generalize to routine clinical practice or to prodromal and advanced disease stages, and external prospective validation in broader cohorts is required.

Given these limitations, the LBSS is offered as a simple and pragmatic first step and illustration of the utility of composite measures in LBD. Future refinement should explore data‐driven methods as above, or stakeholder‐informed weighting and clinimetric approaches to optimize sensitivity and consider incorporation of additional clinical domains that may impact patients and their caregivers such as sleep–wake disturbance and autonomic dysfunction to capture the full multidimensional burden of LBD. This is being pursued through the development of much needed LBD‐specific scales in the form of the Lewy Body Dementia–Domain Rating Scale (LBD‐DRS)^38^ and our data provides support for its premise. External validation in independent datasets, including observational cohorts, clinical trial populations, and real‐world clinical settings across different cultural contexts, will be important to establish generalizability across disease stages and health‐care systems.

In summary, our study provides proof of concept that a simple, clinically grounded composite index can sensitively detect disease progression and intervention effects in LBD over short intervals. By capturing the multidimensional nature of the disorder, the LBSS offers efficiency gains and greater clinical meaningfulness than single‐domain scales. Composites will not replace symptom‐specific endpoints where appropriate, but for DMTs and interventions targeting multiple domains, they represent a crucial methodological advance. Our findings support the refinement of multidomain scales in LBD and suggest that a composite index may serve as a timely and pragmatic candidate as a clinical endpoint for therapeutic trials while disease‐specific scales are being validated and developed.

## AUTHOR CONTRIBUTIONS

**Elie Matar**: Research project; conception; organization; execution; statistical analysis; design; execution; review and critique; manuscript preparation; writing of the first draft; review and critique. **Simon R. White**: Statistical analysis; design; execution; review and critique; manuscript preparation; review and critique. **John‐Paul Taylor**: Research project; organization; statistical analysis; review and critique; manuscript preparation; review and critique. **Alan Thomas**: Research project; conception; organization; statistical analysis; review and critique; manuscript preparation; review and critique. **Ian McKeith**: Research project; conception; organization; manuscript preparation; review and critique. **Joseph Kane**: Research project; execution; statistical analysis; review and critique; manuscript preparation; review and critique. **Simon Lewis**: Statistical analysis; review and critique; manuscript preparation; review and critique. **Ajenthan Surendranathan**: Research project; organization; execution; statistical analysis; review and critique; manuscript preparation; review and critique. **John O'Brien**: Research project; conception; organization; execution; statistical analysis; review and critique; manuscript preparation; review and critique.

## CONFLICT OF INTEREST STATEMENT

The authors have no relevant conflicts of interest to disclose. Author disclosures are available in the supporting information.

## Supporting information



Supporting Information

Supporting Information

## References

[1] Emre M , Aarsland D , Brown R , Burn DJ , Duyckaerts C , Mizuno Y , et al. Clinical diagnostic criteria for dementia associated with parkinson's disease. Mov Disord. 2007;22:1689–1707. 17542011 10.1002/mds.21507

[2] McKeith IG , Boeve BF , Dickson DW , Halliday G , Taylor JP , Weintraub D , et al. Diagnosis and management of dementia with Lewy bodies: fourth consensus report of the DLB consortium. Neurology. 2017;89:88–100. 28592453 10.1212/WNL.0000000000004058 PMC5496518

[3] Mastenbroek SE , Vogel JW , Collij LE , Serrano GE , Tremblay C , Young AL , et al. Disease progression modelling reveals heterogeneity in trajectories of Lewy‐type α‐synuclein pathology. Nature Communications. 2024;15:5133. 10.1038/s41467-024-49402-x PMC11180185 38879548

[4] Inguanzo A , Poulakis K , Mohanty R , Schwarz CG , Przybelski SA , Diaz‐Galvan P , et al. MRI data‐driven clustering reveals different subtypes of Dementia with Lewy bodies. NPJ Parkinson's Disease. 2023;9:5. 10.1038/s41531-023-00448-6 PMC9859778 36670121

[5] Abdelnour C , Ferreira D , van de Beek M , Cedres N , Oppedal K , Cavallin L , et al. Parsing heterogeneity within dementia with Lewy bodies using clustering of biological, clinical, and demographic data. Alzheimers Res Ther. 2022;14:14. 35063023 10.1186/s13195-021-00946-w PMC8783432

[6] Gharbi A , Nasri A , Sghaier I , Kacem I , Mrabet S , Souissi A , et al. Subtypes of Dementia with Lewy bodies: clinical features, survival, and apolipoprotein E effect. J Alzheimers Dis Rep. 2023;7:1277–1288. 38143772 10.3233/ADR-230064 PMC10741894

[7] Choudhury P , Graff‐Radford J , Aakre JA , Wurtz L , Knopman DS , Graff‐Radford NR , et al. The temporal onset of the core features in dementia with Lewy bodies. Alzheimers Dement. 2022;18:591–601. 34761850 10.1002/alz.12411 PMC8986606

[8] Rodriguez‐Porcel F , Wyman‐Chick KA , Abdelnour Ruiz C , Toledo JB , Ferreira D , Urwyler P , et al. Clinical outcome measures in dementia with Lewy bodies trials: critique and recommendations. Transl Neurodegener. 2022;11:24. 35491418 10.1186/s40035-022-00299-w PMC9059356

[9] Goetz CG , Tilley BC , Shaftman SR , Stebbins GT , Fahn S , Martinez‐Martin P , et al. Movement disorder society‐sponsored revision of the unified Parkinson's disease rating scale (MDS‐UPDRS): scale presentation and clinimetric testing results. Mov Disord. 2008;23:2129–2170. 19025984 10.1002/mds.22340

[10] Abdelnour C , Gonzalez MC , Gibson LL , Poston KL , Ballard CG , Cummings JL , et al. Dementia with Lewy bodies drug therapies in clinical trials: systematic review up to 2022. Neurol Ther. 2023;12:727–749. 37017910 10.1007/s40120-023-00467-8 PMC10195935

[11] Sims JR , Zimmer JA , Evans CD , Lu M , Ardayfio P , Sparks J , et al. Donanemab in early symptomatic Alzheimer disease: the TRAILBLAZER‐ALZ 2 randomized clinical trial. Jama. 2023;330:512–527. 37459141 10.1001/jama.2023.13239 PMC10352931

[12] Pagano G , Taylor KI , Anzures Cabrera J , Simuni T , Marek K , Postuma RB , et al. Prasinezumab slows motor progression in rapidly progressing early‐stage Parkinson's disease. Nat Med. 2024;30:1096–1103. 38622249 10.1038/s41591-024-02886-y PMC11031390

[13] McMenamin M , Berglind A , Wason JMS . Improving the analysis of composite endpoints in rare disease trials. Orphanet J Rare Dis. 2018;13:81. 29788976 10.1186/s13023-018-0819-1 PMC5964664

[14] Schneider LS , Goldberg TE . Composite cognitive and functional measures for early stage Alzheimer's disease trials. Alzheimers Dement (Amst). 2020;12:e12017. 32432155 10.1002/dad2.12017 PMC7233425

[15] Estevez‐Fraga C , Scahill RI , Durr A , Leavitt BR , Roos RAC , Langbehn DR , et al. Composite UHDRS correlates with progression of imaging biomarkers in Huntington's disease. Mov Disord. 2021;36:1259–1264. 33471951 10.1002/mds.28489

[16] Jaeger J , Yang L , Li Y , Castrillo‐Viguera C , Haeberlein SB , Dam T , et al. Development of a cognitive composite for measuring change in progressive supranuclear palsy. Parkinsonism Relat Disord. 2021;92:94–100. 34736158 10.1016/j.parkreldis.2021.10.007

[17] Boeve BF , Rosen H , Boxer A , Kornak J , Heuer H , Fields J , et al. The multidomain impairment rating (MIR) scale: initial reliability data on a multidimensional scale for FTLD. Neurology. 2019;92(suppl 15):P5.1‐010.

[18] Poos JM , Moore KM , Nicholas J , Russell LL , Peakman G , Convery RS , et al. Cognitive composites for genetic frontotemporal dementia: GENFI‐Cog. Alzheimers Res Ther. 2022;14:10. 35045872 10.1186/s13195-022-00958-0 PMC8772227

[19] Matar E , White SR , Taylor JP , Thomas A , McKeith IG , Kane JPM , et al. Progression of clinical features in Lewy body dementia can be detected over 6 months. Neurology. 2021;97:e1031–e40. 34404743 10.1212/WNL.0000000000012450 PMC8448556

[20] O'Brien JT , McKeith IG , Thomas AJ , Bamford C , Vale L , Hill S , et al. Introduction of a management toolkit for Lewy body dementia: a pilot cluster‐randomized trial. Mov Disord. 2021;36(1):143‐151. 32960456 10.1002/mds.28282

[21] McKeith IG , Dickson DW , Lowe J , Emre M , O'Brien JT , Feldman H , et al. Diagnosis and management of dementia with Lewy bodies: third report of the DLB consortium. Neurology. 2005;65:1863–1872. 16237129 10.1212/01.wnl.0000187889.17253.b1

[22] Lee DR , McKeith I , Mosimann U , Ghosh‐Nodial A , Grayson L , Wilson B , et al. The dementia cognitive fluctuation scale, a new psychometric test for clinicians to identify cognitive fluctuations in people with dementia. Am J Geriatr Psychiatry. 2014;22:926–935. 24332982 10.1016/j.jagp.2013.01.072

[23] Cummings JL , Mega M , Gray K , Rosenberg‐Thompson S , Carusi DA , Gornbein J . The neuropsychiatric inventory: comprehensive assessment of psychopathology in dementia. Neurology. 1994;44:2308–2314. 7991117 10.1212/wnl.44.12.2308

[24] Del Ser T , McKeith I , Anand R , Cicin‐Sain A , Ferrara R , Spiegel R . Dementia with lewy bodies: findings from an international multicentre study. Int J Geriatr Psychiatry. 2000;15:1034–1045. 11113984 10.1002/1099-1166(200011)15:11<1034::aid-gps231>3.0.co;2-5

[25] Bucks RS , Ashworth DL , Wilcock GK , Siegfried K . Assessment of activities of daily living in dementia: development of the bristol activities of daily living scale. Age and Ageing. 1996;25:113–120. 8670538 10.1093/ageing/25.2.113

[26] Zarit SH , Reever KE , Bach‐Peterson J . Relatives of the impaired elderly: correlates of feelings of burden. The Gerontologist. 1980;20:649–655. 7203086 10.1093/geront/20.6.649

[27] Emre M , Aarsland D , Albanese A , Byrne EJ , Deuschl G , De Deyn PP , et al. Rivastigmine for dementia associated with Parkinson's disease. N Engl J Med. 2004;351:2509–2518. 15590953 10.1056/NEJMoa041470

[28] Mori E , Ikeda M , Kosaka K , Donepezil DLBSI . Donepezil for dementia with Lewy bodies: a randomized, placebo‐controlled trial. Ann Neurol. 2012;72:41–52. 22829268 10.1002/ana.23557 PMC3504981

[29] Ikeda M , Mori E , Kosaka K , Iseki E , Hashimoto M , Matsukawa N , et al. Long‐term safety and efficacy of donepezil in patients with dementia with Lewy bodies: results from a 52‐week, open‐label, multicenter extension study. Dement Geriatr Cogn Disord. 2013;36:229–241. 23949147 10.1159/000351672

[30] McKeith I , Del Ser T , Spano P , Emre M , Wesnes K , Anand R , et al. Efficacy of rivastigmine in dementia with Lewy bodies: a randomised, double‐blind, placebo‐controlled international study. Lancet. 2000;356:2031–2036. 11145488 10.1016/S0140-6736(00)03399-7

[31] Tariot PN , Cummings JL , Soto‐Martin ME , Ballard C , Erten‐Lyons D , Sultzer DL , et al. Trial of pimavanserin in dementia‐related psychosis. N Engl J Med. 2021;385:309–319. 34289275 10.1056/NEJMoa2034634

[32] Weintraub D , Espay AJ , Sharma VD , Tariot PN , Abler V , Pathak S , et al. Pimavanserin for psychosis in Parkinson's disease dementia: subgroup analysis of the HARMONY trial. Parkinsonism Relat Disord. 2024;119:105951. 38113700 10.1016/j.parkreldis.2023.105951

[33] Murata M , Odawara T , Hasegawa K , Iiyama S , Nakamura M , Tagawa M , et al. Adjunct zonisamide to levodopa for DLB parkinsonism: a randomized double‐blind phase 2 study. Neurology. 2018;90:e664–e672. 29367449 10.1212/WNL.0000000000005010 PMC5818167

[34] Wang J , Logovinsky V , Hendrix SB , Stanworth SH , Perdomo C , Xu L , et al. ADCOMS: a composite clinical outcome for prodromal Alzheimer's disease trials. J Neurol Neurosurg Psychiatry. 2016;87:993–999. 27010616 10.1136/jnnp-2015-312383 PMC5013117

[35] Cohen S , Cummings J , Knox S , Potashman M , Harrison J . Clinical trial endpoints and their clinical meaningfulness in early stages of Alzheimer's disease. J Prev Alzheimers Dis. 2022;9:507–522. 35841252 10.14283/jpad.2022.41 PMC9843702

[36] Buracchio T , Campbell M , Krudys K . Assessing clinical meaningfulness in clinical trials for Alzheimer's disease: A U.S. regulatory perspective. Alzheimers Dement (N Y) 2025;11:e70113. 40469853 10.1002/trc2.70113 PMC12136089

[37] Kane JPM , Fitzpatrick RL , Betzhold S , Daly G , Kalfas E , Kinchin I , et al. A common outcome set for trials in dementia with Lewy bodies (DLB COS). Alzheimers Dement (N Y). 2025;11:e70134. 40657378 10.1002/trc2.70134 PMC12254044

[38] Matar E , Boeve B , Kane J , Rodriguez‐Porcel F , Schrag A , Sikkes SA , et al. Development of the Lewy body dementia domain rating scale (LBD‐DRS): a disease‐specific outcome measure. Mov Disord. 2025;40(suppl 1).

