# Supplementary material for: The utility of a composite endpoint for tracking disease progression in Lewy body dementia
Source: Alzheimers Dement (N Y). 2026 May 20;12(2):e70260. doi: 10.1002/trc2.70260 (PMC13239424; doi:10.1002/trc2.70260)
Supplement: Supplementary file 1 — Supporting Information [file TRC2-12-e70260-s001.docx]

**Supplementary Material**

**Table of Contents**

**S1. Standardized Lewy Body Symptom Severity Index (LBSSz)
 S1.1**Construction of the Standardized LBSS (LBSSz) **S1.2**Demographics of LBSSz **S1.3**Longitudinal Change Over Time in LBSSz and Its Constituent Components **S1.4**Discussion of the LBSSz and Its Constituent Components **S1.5**Example Method for Optimization of Weights Using the Standardized LBSS (LBSSz)
**S1.6**Optimization of Weights Using the Standardized LBSS (LBSSz) and Comparison to Pre-optimized LBSSz

**S2. Individual Domain Trajectories Over Time**

**Supplementary Table 1.**Linear mixed-effects model estimates of longitudinal change (time) and intervention effects (Intervention × Time) for the standardized LBSSz and its components

**S1. Standardized LBSS (LBSSz)**

**S1.1 Construction of the Standardized LBSS (LBSSz)**

To enable a multidomain assessment of progression in Lewy body dementia (LBD), we constructed the Lewy Body Symptom Severity Index (LBSS) from established clinical measures spanning the core symptomatic domains of the disease. Cognitive performance was captured using the Mini-Mental State Examination (MMSE), motor parkinsonism was assessed with the Unified Parkinson’s Disease Rating Scale part III (UPDRS-III), cognitive fluctuations were measured with the Dementia Cognitive Fluctuation Scale (DCFS), and neuropsychiatric symptoms were indexed using the Neuropsychiatric Inventory 4-item subscale (NPI-4, comprising hallucinations, delusions, apathy, and depression).

Following the above, a standardized version (LBSS_z_) was constructed as an alternative model to ensure that the directions are consistent. Each constituent scale was transformed into a z-score anchored to the distribution of the baseline cohort (mean = 0, standard deviation = 1 across all participants at study entry). The standardized component scores were then combined with equal weighting to generate the composite LBSSz:

$$\mathrm{LBSS}_{z}= -\mathrm{MMSE}_{z}+{\mathrm{UPDRS}\text{-}\mathrm{III}}_{z}+\mathrm{DCFS}_{z}+{\mathrm{NPI}\text{-4}}_{z}$$

This approach ensured that differences in measurement range and variance across the contributing scales were accounted for, while also allowing longitudinal changes to be interpreted relative to the baseline variance of the study population. To measure progression over time, a linear mixed effect model was constructed equivalent to the rescaled score (see Methods section in the main manuscript). Fixed covariates in all models included age, diagnosis (DLB or PDD), antipsychotic use, cholinergic medication use, and levodopa equivalent dose. Time was coded as months since baseline (0, 3, and 6). The model included random intercepts for participants to account for repeated measures.

**S1.2 Demographics of LBSSz**

The standardized LBSS_z_ values were highly consistent across groups (DLB: 0.79 ± 0.06; PDD: 0.80 ± 0.06; total LBD cohort: 0.79 ± 0.06). Overall, the LBSS_z_ distribution was tightly clustered around the mean with relatively small variance.

**S1.3 Longitudinal change over time of LBSSz and its constituent components**

Over the study period, the standardized LBSS_z_ demonstrated a significant increase with time (β = +0.0041, SE = 0.0014, p = 0.0046), reflecting progressive multidomain symptom worsening across the cohort (Supplementary Table 1). Among the individual components, motor parkinsonism (UPDRS-III_z_; β = +0.83, p = 0.010) and cognitive fluctuations (DCFS_z_; β = +0.061, p = 0.003) showed significant progression, while global cognition (MMSE_z_) declined modestly without reaching statistical significance (β = −0.041, p = 0.073), and neuropsychiatric symptoms (NPI-4_z_) did not change significantly (p = 0.290). Taken together, these findings indicate that the LBSS_z_ was sensitive to short-term progression in LBD, capturing changes not consistently observed with individual domain measures.

**S1.4 Discussion regarding the LBSSz and its constituent components**

Analysis of the standardized LBSSz produced findings that were directionally consistent with the simpler raw composite, suggesting that the composite retained robust properties across different construction methods. However, because the standardized scores were normalized to the study cohort, their generalizability is limited and the additional complexity was not justified. For these reasons, the simpler raw score was preferred for subsequent analyses, including power estimation and derivation of the minimal clinically important difference (MCID) as outlined in the main text.

**S1.5 Example method for the optimization of weights using the standardized LBSS (LBSSz)**

As an illustration for deriving empirically optimized weights for the LBSSz, a constrained nonlinear optimization procedure was implemented in MATLAB (R2023b, MathWorks Inc.) using standardized (z-scored) component measures from above: UPDRS-III_z_, MMSE_z_, DCFS_z_, and NPI-4_z_. The goal was to identify the linear combination of these domains that maximized sensitivity to longitudinal change and minimizing the statistical power required to detect change over time using the LBSSz.

For each candidate weighting vector $w=\left( w_{1},w_{2},w_{3},w_{4} \right)$ a composite score was computed as:

$$\mathrm{LBSS}_{z}= -{w_{1}\cdot\mathrm{MMSE}}_{z}+{w_{2}\cdot UPDRS\text{-}\mathrm{III}}_{z}+w_{3}{\cdot DCFS}_{z}+{w_{4}\cdot NPI\text{-4}}_{z}$$

A linear mixed-effects model was then fitted using the MATLAB function *fitlme* for each weighted composite, with equivalent fixed effects and random intercepts as per the models used above:

$${LBSS}_{z}=\beta_{0}+\beta_{1}\cdot Time+\beta_{2}\cdot diagnosis+\beta_{3}\cdot age+\beta_{4}\cdot LED+\beta_{5}\cdot Cholinergic dose + \beta_{6}\cdot Antipsychotic use + \beta_{7}\cdot Intervention\times Time +(Random Intercept)+ \varepsilon$$

The optimization objective was to maximize the absolute standardized coefficient for time $\left( \left| \beta_{1} \right| \right)$ which reflects responsiveness to disease progression, while minimizing model residual variance (approximated by the Bayesian Information Criterion, BIC). The combined objective function was:

$$\min_{w} \mathcal{L(}w)=\left( \left| \beta_{1}(w) \right| \right)+BIC(w)$$

subject to the constraints: $w\geq0$ and $\sum_{i-=1}^{4} w_{i} =1$

Optimization was performed using MATLAB’s *fmincon* function initialized with equal weights ($w_{i} =0.25)$. The resulting optimized weights defined the final LBSSz composite, providing the most efficient and sensitive measure of short-interval progression across the Lewy body dementia cohort, with the aim of enhancing statistical power for clinical trial endpoints.

**S1.6 Optimization of weights using the Standardized LBSS (LBSSz) and comparison to pre-optimized LBSSz**

Empirical optimization of the LBSSz as above yielded the following weights for the component measures: MMSE = 0.35, DCFS-Total = 0.27, UPDRS-III = 0.27, and NPI-4 = 0.11 (weights constrained to sum to 1). This weighting scheme maximized the longitudinal coefficient for time while minimizing the Bayesian Information Criterion (BIC), thereby enhancing both sensitivity to change and model parsimony. The resulting composite placed greatest emphasis on cognitive and functional domains, followed by motor and neuropsychiatric features, consistent with clinical patterns of short-interval progression in Lewy body dementia.

When evaluated in the linear mixed-effects model, the optimized composite demonstrated a significantly larger longitudinal effect of time (β = 0.030 ± 0.012, p = 0.013) compared with the unoptimized version (β = 0.004 ± 0.001, p = 0.005), representing roughly a seven-fold increase in standardized sensitivity to change. Between-subject variance increased from 0.003 to 0.096, indicating an expanded dynamic range and improved discrimination of individual trajectories. The model remained well-fitted (AIC = 160.5, BIC = 196.4).

Together, these results confirm that empirical weighting (0.35 MMSE + 0.27 DCFS + 0.27 UPDRS + 0.11 NPI) substantially improved the responsiveness of the LBSS composite, providing a more efficient endpoint for detecting short-interval progression and reducing the statistical power required for longitudinal change analyses in Lewy body dementia.

**S2. Results showing individual domain trajectories over time**

Individual domain trajectories were calculated as per prior work^18^. Covarying for cholinergic medication use, MMSE showed a significant decline over time (β=−0.2226, *p*=0.0275), indicating progressive cognitive deterioration. Motor severity, as measured by the MDS-UPDRS-III, increased significantly (β=0.5346, *p*=0.0120), as did the frequency and severity of cognitive fluctuations on the DCFS (β=0.2091, *p*=0.0215). In contrast, the NPI-4 subscore showed no meaningful change over the study period (*p*=0.993).

**Supplementary Table 1**

| Outcome | β (Time) | SE | p-value | AIC | BIC |
| --- | --- | --- | --- | --- | --- |
| *Time* |  |  |  |  |  |
| MMSE**_z_** | −0.041 | 0.023 | 0.073 | 467.2 | 496.7 |
| UPDRS-III**_z_** | +0.825 | 0.319 | 0.010* | 2429.3 | 2462.7 |
| DCFS**_z_** | +0.061 | 0.020 | 0.003** | 479.9 | 510.0 |
| NPI-4**_z_** | +0.205 | 0.193 | 0.290 | 1364.9 | 1398.1 |
| **LBSS_z_** | +0.0041 | 0.0014 | 0.0046** | −577.2 | −542.1 |

Linear mixed model estimates of longitudinal change (time) and intervention effects (Intervention × Time) for the standardized Lewy Body Symptom Severity Index (LBSSz) and its components. β values represent fixed-effect estimates of the slope (mean change per unit time) for each outcome, with associated standard error (SE) and p-values. ‘Time’ reflects the average trajectory across all participants. AIC (Akaike Information Criterion) and BIC (Bayesian Information Criterion) are reported as measures of relative model fit (lower the better). Significance levels are indicated as *p < 0.05, **p < 0.01, ***p < 0.001.
